# Identification of differentially expressed genes in ileal Peyer's Patch of scrapie-infected sheep using RNA arbitrarily primed PCR

**DOI:** 10.1186/1746-6148-4-12

**Published:** 2008-03-28

**Authors:** Lars Austbø, Andreas Kampmann, Ulf Müller-Ladner, Elena Neumann, Ingrid Olsaker, Grethe Skretting

**Affiliations:** 1 Department of Basic Sciences and Aquatic Medicine, Norwegian School of Veterinary Science, P.O. Box 8146 Dep., N-0033, Oslo, Norway; 2 Department of Internal Medicine and Rheumatology, Justus-Liebig-University Gießen, Kerckhoff-Klinik Bad Nauheim, Germany; 3 Hematological Research Laboratory, Ullevål University Hospital, 0407 Oslo, Norway

## Abstract

**Backgound:**

In scrapie and prion diseases, the knowledge concerning genes involved in host response during the early infection period in the lymphoid tissues, still remains limited. In the present study, we have examined differential gene expression in ileal Peyer's patches and in laser microdissected follicles of sheep infected with scrapie.

**Methods:**

Ileal Peyer's patches and laser microdissected follicles were of scrapie and control lambs with susceptible genotypes for classical scrapie. Potential regulated genes were found using RNA arbitrarily primed polymerase chain reaction (RAP-PCR) and fingerprinting. The differentially expressed genes were confirmed using real-time RT-PCR.

**Results:**

The expression of three genes (*MAPRE3, LOC729073 *and *DNAJC3*), were found to be significantly altered in scrapie infected lambs (P < 0.05).

**Conclusion:**

The three genes have not previously been associated with prion diseases and are interesting as they may reflect biological processes involved in the molecular pathogenesis of prion diseases.

## Background

Prion diseases or transmissible spongiform encephalopathies (TSEs), constitute a group of fatal neurodegenerative diseases that occur in a variety of species including sheep (scrapie), cattle (bovine spongiform encephalopathy (BSE)) and humans (e.g. Creutzfeld-Jakob disease (CJD)) [1]. Typical of TSEs is the accumulation of an abnormal protease-resistant form of the cellular prion protein (PrP^C^) named PrP^Sc^[2]. At the terminal stage of the disease, PrP^Sc ^accumulates in the central nervous system (CNS) [3], a prerequisite for the clinical signs and neuropathologic disorders [4,5].

The natural route of infection is considered to be the uptake of infectious agent from the alimentary tract [6]. Initially, and without any sign of normal immune response, PrP^Sc ^accumulation is observed in the peripheral lymphoid tissues [7] where lymphoid follicles are reported to be the first site of accumulation of PrP^Sc^ in both sheep and mice [8-10]. In young sheep a continuous aggregation of lymphoid follicles named the ileal Peyer's patch (PP) is a major component of the gut-associated lymphoid tissue (GALT) [11]. It has been documented that the ileal PP is responsible for the generation of the majority of B-cells and the diversification of the pre-immune antibody repertoire [12,13]. The ileal PP and GALT are interesting in relation to prion diseases since these tissues represent the first site for accumulation of PrP^Sc^. Additionally, in sheep, cattle and humans, a correlation between susceptibility to prion disease and the development of PP has been described [14]. A previous study by our group indicated an upregulation of prion mRNA expression in ileal PP follicles with accumulated PrP^Sc^[15], leading to the assumption that additional genes could be differentially expressed.

There is considerable uncertainty about the biological function of PrP^C ^as well as the molecular mechanisms involved in the uptake and conversion of the scrapie agent. An approach to explore the underlying molecular mechanisms is to identify differentially expressed mRNAs. In the present study, the RNA arbitrarily primed PCR (RAP-PCR) and fingerprint approaches were chosen to screen for differentially expressed genes in the ileal PP of lambs eight months after experimental oral infection with scrapie. RAP-PCR has proven to be an efficient method to detect new genes from laser dissected tissues where only small amounts of RNA are available [16,17]. Basically, cDNAs are synthesised using an arbitrary primer and total RNA from two experimental samples. The cDNA is then amplified using a second arbitrary primer to create a unique fingerprint for each sample. The fragments are labelled with radioactivity and separated on a gel. Differentially expressed genes are identified as bands displaying differences in intensity between the two samples. Desired bands are excised from the gel and cloned for further studies.

Little is known concerning the effects the accumulation of the scrapie agent might have on gene expression in the lymphoid tissues during the early infection period. Genes differentially expressed between scrapie infected and control animals might aid to answer these questions, and could provide the possibility of early identification of the infection and of disease monitoring. Through RAP-PCR, fingerprint screening and verification by real-time RT-PCR, we identified three genes not previously correlated with TSEs, as differentially expressed in lymphoid tissues of sheep infected with scrapie.

## Methods

### Animals

The sheep investigated were of the Norwegian white breed Rygja. Lambs with susceptible genotypes for classical scrapie (AVRRQQ and VVRRQQ) were infected orally as previously described at an age of six to eight weeks with brain homogenate from sheep with scrapie [9]. Age- and genotype-matched control lambs received an oral dose of physiological saline on the same day and in the same manner as the lambs exposed to the scrapie agent. Eight months after exposure to the scrapie agent, the eight inoculated lambs and six matched control lambs were necropsied. Parallel tissue samples were collected for cryostat sectioning and total RNA isolation. Samples collected for sectioning and laser microdissection were snap frozen in 1,1,1-trifluorethane/1,1,1,2 tetrafluorethane/pentafluorethane (R404A; Ausimont, Bollate, Italy), chilled in liquid nitrogen and stored at -80°C until use. Tissue samples collected for RNA isolation using RNeasy Midi kit were embedded in RNAlater (Ambion, Austin, USA) and stored at -20°C. PrP^Sc ^accumulation was verified using immunohistochemistry. All the animals exposed to scrapie have previously been described [15] and had accumulation of PrP^Sc ^in ileal PP follicles. All matching control lambs were found negative for PrP^Sc ^by the same procedure.

### Ethical aspects and safety provisions

The experimental inoculations with scrapie-infected material were conducted in the confined and controlled isolation facilities of the Norwegian School of Veterinary Science in Sandnes, Norway. Legal and ethical national requirements and code of practice were implemented in the animal experiments.

### Laser capture microdissection

Sections of 14 μm thickness were cut using a cryostat (Leitz Cryostat 1720, Wetzlar, Germany) and mounted on special membrane slides for laser microdissection (Molecular Machines & Industries, Glattbrugg, Switzerland). The sections were air-dried at room temperature and stored at -80°C until use. Prior to microdissection, the membrane sections were stained with RNase free hematoxylin and dried at room temperature. Laser capture microdissection of tissue sections was performed using the SLμCut laser microdissection system (Molecular Machines & Industries). To ensure that the material was representative and sufficient, several follicles were microdissected, i.e. up to 50 different follicles from each animal were used for the RAP-PCR and fingerprint experiments.

### RNA extraction

Total RNA from laser-captured tissue was isolated using the Absolutely RNA Nanoprep kit (Stratagene, Amsterdam, The Netherlands). The kit allows rapid purification of high-quality total RNA from extremely small samples of cells (1 × 10^4 ^cells) harvested by laser capture microdissection with an expected yield up to 100 ng. The manufacturer's protocol was followed including the optional DNase step. The RNA was eluted into 20 μl elution buffer, and stored at -80°C. Total RNA from ileal PP tissue was isolated using approximately 100 mg of tissue and RNeasy Midi Kit (Qiagen, GmbH, Germany). The RNA was eluted into 150 μl elution buffer, and stored at -80°C

### RAP-PCR fingerprinting

RAP-PCR was carried out according to the method of Judex et al. with some modifications [18]. Briefly, first strand cDNA synthesis was carried out using OPN 21 primers (20 μM) (Table 1) and 50–100 ng of total RNA in a final volume of 30 μl that included 10 U Mu-MLV reverse transcriptase, 1 × RT buffer (Eurogentec, Seraing, Belgium), 0.2 mM dNTP and 30 U RNase inhibitor (Eurogentec). Following a 5 min ramp from 27°C to 37°C the reaction was carried out at 37°C for 60 min. Inactivation of reverse transcriptase was performed at 68°C for 15 min. Subsequently, the cDNA was split into 10 PCR reactions with different arbitrarily primers (Table 1). The PCR reactions were carried out in a volume of 20 μl consisting of 2 U Taq DNA Polymerase, 1× buffer (Qiagen), 0.2 mM dNTP, 0.4 μM primer and 0.2 μCi [alpha-^32^P dCTP] (Amersham Biosciences, Braunschweig, Germany). The amplification conditions were 30 cycles at 94°C for 30 seconds, 35°C for 30 seconds and 72°C for 60 seconds. An aliquot of the PCR product was mixed with loading dye and denatured at 95°C before loading onto a 5% polyacrylamide gel (5 M urea/TBE buffer), which was run at 100 W (45°C) for approx. 90 min. After drying under vacuum at 80°C for 1–2 h, the gel was exposed to a Hyperfilm™ x-ray film (GE Healthcare, Buckinghamshire, UK). Differentially expressed bands were excised from the gel and soaked in water for 10 min before re-amplification using the same primers and PCR conditions. Prior to the RAP-PCR, different combinations of reverse transcription and PCR primers were tested to achieve the combinations that gave the best span in fragment sizes. For all primer combinations a non-DNA sample was included to rule out the presence of any unwanted bands caused by primer dimers or contamination.

**Table 1:** Primers used for reverse transcription (RT), RAP-PCR and verification by real-time PCR.

**NAME**	**PRIMER**
RT primer	
OPN21	ACCAGGGGCA
**RAP-PCR primers**	
Br2	ACTCGTAAGC
CG2	ACGACGGCGGA
Hurra3	CACCCGTAGTC
KinaseA+	GAGGGTGCCTT
MJ3	CCAATTCAGTT
MJ4	GAGATGACGACC
Nuclear1-	TTGCTCTTCTT
Nuclear2-	CTCTTCTTGGT
OPN23	CAGGGGCACC
US6	GTGGTGACAG
**Real-time primers**	
GAPDH F	TGATTCCACCCATGGCAAGT
GAPDH R	CCACGTACTCAGCACCAGCAT
DNAJC3 F	GTGGAGCTAATAGGAAAAACATTCC
DNAJC3 R	AATGAGTTGCTTGTCTGTCTTTATTC
CG6405-PA F	CCAGACAAACTGGGCTTTAGC
CG6405-PA R	ATGTGCAAGGCACTTAGTACTGG
MAPRE3 F	GCTTTCAAGAAGATGGGTGTTGAC
MAPRE3 R	TTTGTTGAAGATCTGATCACCTGG

### Cloning and sequencing

The re-amplified RAP-PCR bands were cloned into TOPO vector using the TOPO – TA cloning kit (Invitrogen, De Scelp, The Netherlands). Sequence analysis of the fragments was performed using dye-labelled nucleotides (DYEnamic ET DYE terminator Kit, GE Healthcare) on a MegaBACE 1000 sequencer (GE Healthcare). Because one RAP-PCR band could represent several genes, 8 – 12 parallel colonies were sequenced and the sequence in majority were analyzed further for homologies to known sequences using BLAST sequence similarity searching network service at the National Center for Biotechnology Information [19].

### Verification of differentially expressed genes by quantitative Real-Time RT-PCR

Quantitative real-time RT-PCR was performed using a one-step Ampliqon RealQ PCR Master mix kit with Green DNA dye included (Ampliqon, Herlev, Denmark) and 2.5 U Mu-MLV reverse transcriptase (Eurogentec,) in 25 μl reaction volume. Primers were designed using PrimerExpress 1.5 (Applied Biosystems, Foster City, USA). The expression level was measured with relative quantification using glyceraldehyde-3-phosphate dehydrogenase (*GAPDH*) as the reference gene on all samples. Each quantification target was amplified in triplicate samples and negative control lacking RT enzyme was always included in the experiments to ensure for any genomic contamination. Primers for quantitative real-time RT-PCR are presented in Table 1. Real-time RT-PCR was carried out in a Chromo 4 (Bio-Rad, Hercules, USA) using the following uniform temperature profile: 30 min at 50°C (reverse transcription), then 15 min at 95°C (denaturation), followed by 40 cycles of 30 s at 95°C, 15 s at 58°C and 60 s at 72°C. The same cycling profile was used for all real-time RT-PCRs. The data was analysed using Opticom Monitor fluorescence detection software version 3.1 (MJ Research, MA, USA).

### Statistics

For data sets that were sampled from a Gaussian distribution, statistical differences were evaluated using the non-paired t-test. The distribution of data sets was tested using the Kolmogorov-Smirnov normality test. Differences in expression were considered to be significant with values of probability P < 0.05 (InStat GraphPad Software, San Diego, USA).

## Results

### RAP-PCR fingerprinting

**Figure 1:**
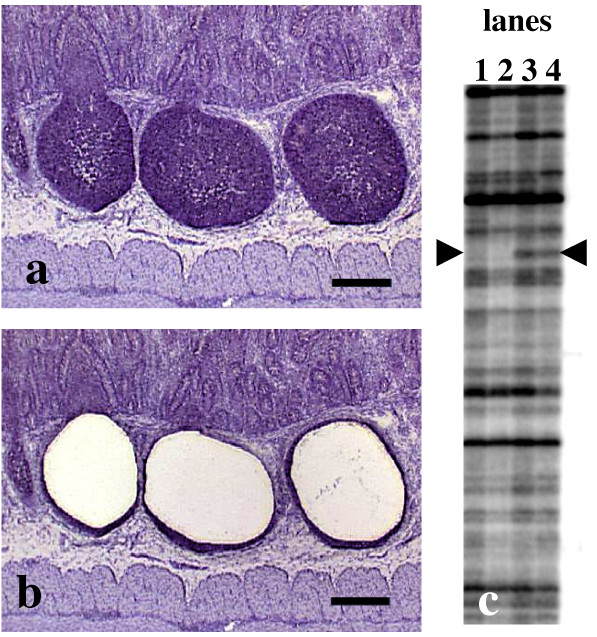
Laser-assisted microdissection of follicles in the ileal PP. Nuclei of the tissue sections were stained with hematoxylin. After laser-assisted dissection of each area (a) the samples were collected (b) using an adhesive membrane and transferred to a cap of a 0.5 ml reaction tube and kept at -20°C until RNA extraction. Bar: 200 μm. (c) A section of RAP-PCR fingerprint showing follicles from control animals (lanes 1–2), and follicles from scrapie animals (lanes 3–4). Arrowheads show a band differentially amplified by RAP-PCR indicating an up-regulation in the follicles of the scrapie infected animals.

For the RAP-PCR experiments, ileal PP from highly susceptible PrP^VRQ/VRQ ^sheep characterized by significant PrP^Sc ^accumulation in peripheral lymphoid tissues and ileal PP from matching control animals with no PrP^Sc ^accumulation were used. The RAP-PCR fingerprint experiment was devided into two parts, one using RNA isolated from ileal PP tissue and the other one using RNA from follicles laser microdissected from ileal PP. In the experiment using RNA isolated from ileal PP tissue, three infected (pooled) and three control animals (pooled) with highly susceptible PrP^VRQ/VRQ ^genotypes were utilized. Duplicates of RAP-PCR reactions were run to exclude the presence of any unwanted variabilities in the PCR amplification. In the second part using laser microdissection, two infected and two control animals with high susceptible PrP^VRQ/VRQ ^genotypes were used. To obtain sufficient amounts of RNA (approx. 50 ng) without performing additional nested PCR, only animals with large follicles were utilized and approx. 50 follicles from each animal were dissected (Fig. 1a and 1b). Since exact measurements of RNA are not feasible for such small amounts, the RNA samples were examined separately and not pooled (Fig. 1c). A total of 25 bands were found differentially amplified by RAP-PCR. Of these were 14 bands from the gel representing ileal PP tissue and 11 bands from the gel representing laser microdissected follicles.

### Sequencing and verification by real-time PCR

The bands differentially amplified were excised from the fingerprint gel, re-amplified, cloned and sequenced. To eliminate any unwanted sequences hiding behind our desired band, 8–12 clones originating from the same band were sequenced. The sequence represented by a majority of the clones was analyzed further. Sequences representing rRNA or with repeated elements were not included in the further studies.

**Figure 2:**
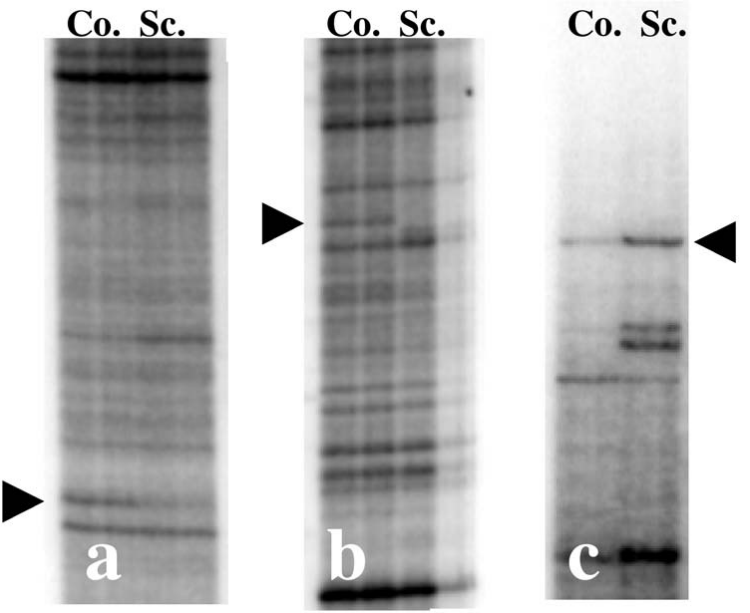
RAP-PCR fingerprints showing the three genes that were verified as differentially expressed in the ileal PP: a) *DNAJC3 *amplified using primer Br2, b) *LOC729073 *amplified using primer Hurra3 and c) *MAPRE3 *amplified using primer OPN23. The lanes represent ileal PP from control animals (Co) and ileal PP from scrapie animals (Sc). Arrowheads indicate the bands of the differentially expressed genes.

A total of 12 potential regulated genes were selected for verification by real-time RT-PCR, nine from total ileal PP tissue and three from laser-dissected follicles. Verification of all the 12 differentially expressed genes was performed by real-time RT-PCR using total RNA isolated from whole ileal PP of 14 lambs; eight scrapie-infected lambs killed eight months after oral exposure with susceptible PrP genotypes (4 PrP^ARQ/VRQ ^and 4 PrP^VRQ/VRQ^) were compared with six controls of the same age and PrP genotype (3 PrP^ARQ/VRQ ^and 3 PrP^VRQ/VRQ^). Additional verification of the three differentially expressed genes in the ileal PP follicles was carried out using total RNA from the laser-dissected follicles originating from the same 14 lambs. For real-time PCR the animal samples were not pooled, but run separately. In total, the expression of three genes was significantly altered in ileal PP of the scrapie-infected animals (Table 2). The fingerprint bands representing these three genes are presented in Figure 2. The first gene is predicted to encode a DnaJ (Hsp40) homolog, subfamily C, member 3 (DNAJC3) and was significantly altered with a threefold higher expression in the uninfected animals (P = 0.02). The second gene LOC729073, which encodes a protein of unknown function, showed strong similarity to several *Bos taurus *and *Ovies aries *ESTs and was significantly altered with a threefold higher expression in the uninfected animals (P = 0.02). The third gene encoding a protein named microtubule-associated protein, RP/EB family, member 3 (MAPRE3, EB3), was significantly altered with a threefold increase in expression in the scrapie-infected animals (P = 0.05). None of the genes, which were found differentially amplified by RAP-PCR fingerprint in the laser dissected ileal PP follicles, were verified as significant regulated (P < 0.05) by real-time PCR.

**Table 2:** Genes found to be regulated in ileal PP of scrapie animals

**Gene symbol**	**Regulation**	**Homology**	**ID**	**Accession**	**region cloned**
*DNAJC3 *(bt)	down	DnaJ (Hsp40) homolog, subfamily C, 3	90%	NW_001493080.1	459249–459497
*LOC729073 *(bt)	down	similar to CG6405-PA	94%	CB535886	8–364
*MAPRE3 *(bt)	up	microtubule-associated protein, RP/EB family, 3	98%	NM_001075917	282–587

Sequences were compared using NCBI Blastn [19]. The highest identity of the longest fragment matching with the database sequence is shown. (bt) bovine.

The *GAPDH *expression in the ileal PP were evaluated relative to the amount of RNA and showed stable expression among the different animals and experimental groups (data not shown). As for the laser dissected follicles, the total amount of RNA obtained (< 50 ng) was too small to allow accurate measurements of the RNA concentrations making it impossible to perform an evaluation of *GAPDH*. Consequently, the data obtained from the laser dissected follicles had to be based on the assumption that the reference gene, *GAPDH*, was equally expressed in the two groups and not influenced by the scrapie infection.

## Discussion

A useful approach to investigate molecular mechanisms underlying the pathogenesis of scrapie and TSEs is the study of transcriptomes. Recently, various techniques have emerged to compare gene expression profiles in different infected and uninfected models [20]. In the present study we have chosen to use RAP-PCR fingerprinting. Compared to other available methods, this technique has some advantages. It is applicable for all species regardless of accessible genetic data and commercial arrays. Only small amounts of RNA are required to examine specific areas of a tissue obtained by laser microdissection. Common for all RNA fingerprint methods are their potential to compare multiple experimental samples simultaneously and to identify genes that are either up- or down-regulated in one sample relative to another. Some few studies have reported genes differentially regulated in scrapie and TSEs, mostly from brain tissues [21-23]. In a similar study ileal PP of scrapie lambs were examined by cDNA representational difference analysis (RDA) one week after oral inoculation with scrapie agent [24]. At this stage little or no PrP^Sc ^accumulation has been observed in the ileal PP. To analyse the effect of heavy PrP^Sc ^accumulation, we examined ileal PP from lambs eight months after oral inoculation with scrapie using RAP-PCR. Compared to RDA, RAP-PCR has the advantage that this technique allows the analysis of differential gene expression in small RNA samples from laser-dissected follicles. However, one of the current disadvantages of RAP-PCR and other RNA fingerprint based methods are the high number of false positives, and/or gene fragments that seem to be differentially expressed as an artifact of PCR [20]. Consequently, the RAP-PCR fingerprints must be regarded as the first step in an elimination process where all potential differentially expressed genes must be confirmed individually. In the present study we have used real-time PCR on a total of 14 animals to individually confirm the differentially expressed genes. The expression of three genes encoding *MAPRE3, LOC729073 *and *DNAJC3 *were significantly altered in ileal PP of the scrapie-infected animals as confirmed by real-time PCR. The MAPRE3 protein localizes to the cytoplasmic microtubule network and binds a homologue of the adenomatous polyposis coli (APC) [25]. It has been suggested that MAPRE3 is involved in stability and/or extension of microtubules during the first step of the neuronal differentiation [25]. Other microtubule-associated proteins like tau have previously been associated with both amyloid plaques of TSEs, and Alzheimer senile plaques [26,27]. LOC729073 has a predicted similarity to human protein CG6405-PA and contain a domain (DUF1208) of unknown function. The protein DNAJC3 acts as an inhibitor of the interferon-induced, dsRNA-activated protein kinase (PKR), which plays a major role in mediating the interferon response to viral infection. Additionally, DNAJC3 is interesting as other members of the DNAJ family (Hsp40) have been associated with formation and propagation of yeast prions [28].

None of the gene expressions potentially altered in the laser dissected ileal PP follicles were verified as significantly altered by real-time PCR. It is possible that the lack of significance in these laser dissected follicles was due to high variability in PrP^Sc ^accumulation between ileal PP follicles in scrapie-inoculated individuals. We have previously observed variability in PrP^Sc ^accumulation between ileal PP follicles of scrapie-inoculated individuals. This was particularly conspicuous in the ARQ/VRQ (eight months post-inoculation) animals, where there was a huge variability among the animals and among their individual ileal PP follicles [15]. Four of the eight scrapie-infected animals that were used for verification by real-time PCR were of ARQ/VRQ genotype. Furthermore, the follicles used for verification showed major morphological differences both in size and shape between the individual animals, which probably was due to the involution of ileal PP starting at young age.

The expression level of housekeeping genes like *GAPDH *was observed to be relatively stable when comparing samples of the same type of tissue, such as the ileal PP. However, the RNA quantity obtained from the laser-dissected follicles was too low to perform an accurate measurement and the stability of the housekeeping genes in these compartments remains unknown. Consequently, a possibility exists that individual differences might have interfered with our ability to achieve significant results.

One interesting feature of the differentially expressed genes obtained by our experiments was a group of genes related to inflammatory and interferon induction. Although only the expression of *DNAJC3 *among the three interferon related genes was found to be significantly altered in this group of animals, they are worth mentioning as they are consistent with previously published data presenting interferon induced genes differentially regulated in TSEs [23,29]. These genes, namely the interferon-induced protein with tetratricopeptide repeats 1 (IFIT1) and the GTPase very large interferon inducible 1 (GVIN1) were identified as upregulated by RAP-PCR. The association between prions and the immune system is complex. Probably due to tolerance for PrP^Sc^, no immune response has been detected in scrapie or Creutzfeldt-Jakob disease. An induction of interferon-sensitive genes without appreciable interferon synthesis has however, been reported. This induction was strikingly similar to what has been shown in some viral infections, suggesting that the TSE agent might be recognized similar to a foreign virus-like particle [23].

## Conclusion

In summary, we identified three differentially expressed genes not previously associated with prion diseases. Expression of the three genes, *MAPRE3, LOC729073 *and *DNAJC3*, were all found significantly altered in ileal PP of scrapie-infected animals. Although no expression of genes were confirmed as significantly altered in the laser dissected follicles of scrapie infected sheep, the combination of techniques utilized in our study can be useful for insight into gene expression profiles of small histologically defined areas. In this study, ten different arbitrary primers have been tested. Other primer combinations could possibly reveal additional regulated genes. Moreover, beyond the accumulation of PrP^Sc ^seen in the follicles of scrapie-infected animals, there are no pathological characteristics or signs of a normal immune response. These features might reflect that a low number of genes are influenced by PrP^Sc ^accumulation. Further studies are essential to verify the role of these differentially expressed genes in scrapie disease, and particularly important is verification at the protein level and further how these proteins interact in the different pathways. Hopefully these genes will provide additional understanding of the TSE disease process and might in the long-term, lead to the development of new approaches for diagnostics and/or treatment of the disease at an early stage before irreparable damage has occurred.

## Competing interests

The author(s) declare that they have no competing interests.

## Authors' contributions

LA worked on all aspects of the study, design of the project, performed laser microdissection, RAP-PCR fingerprint, qPCR, interpretation of results as well as statistical analysis and writing of the manuscript. AK, UML and EN participated and gave advice on RAP-PCR fingerprint and participated in editing of the manuscript. IO participated in editing of the manuscript. GS participated in design of the project, interpretation of findings and editing of the manuscript. All authors read and approved the final manuscript.

## References

[B1] Johnson RT (2005). Prion diseases. Lancet Neurol.

[B2] Prusiner SB, Scott MR, DeArmond SJ, Cohen FE (1998). Prion protein biology. Cell.

[B3] Caughey B, Raymond GJ, Callahan MA, Wong C, Baron GS, Xiong LW (2001). Interactions and conversions of prion protein isoforms. Adv Protein Chem.

[B4] Detwiler LA, Baylis M (2003). The epidemiology of scrapie. Rev Sci Tech.

[B5] Jeffrey M, Gonzalez L (2004). Pathology and pathogenesis of bovine spongiform encephalopathy and scrapie. Curr Top Microbiol Immunol.

[B6] Hadlow WJ, Kennedy RC, Race RE (1982). Natural infection of Suffolk sheep with scrapie virus. J Infect Dis.

[B7] van Keulen LJ, Schreuder BE, Vromans ME, Langeveld JP, Smits MA (1999). Scrapie-associated prion protein in the gastrointestinal tract of sheep with natural scrapie. J Comp Pathol.

[B8] Andreoletti O, Berthon P, Marc D, Sarradin P, Grosclaude J, van Keulen L, Schelcher F, Elsen JM, Lantier F (2000). Early accumulation of PrP(Sc) in gut-associated lymphoid and nervous tissues of susceptible sheep from a Romanov flock with natural scrapie. J Gen Virol.

[B9] Heggebø R, Press CM, Gunnes G, Lie KI, Tranulis MA, Ulvund M, Groschup MH, Landsverk T (2000). Distribution of prion protein in the ileal Peyer's patch of scrapie-free lambs and lambs naturally and experimentally exposed to the scrapie agent. J Gen Virol.

[B10] Kimberlin RH, Walker CA (1989). Pathogenesis of scrapie in mice after intragastric infection. Virus Res.

[B11] Landsverk T, Halleraker M, Aleksandersen M, McClure S, Hein W, Nicander L (1991). The intestinal habitat for organized lymphoid tissues in ruminants; comparative aspects of structure, function and development. Vet Immunol Immunopathol.

[B12] Gerber HA, Morris B, Trevella W (1986). The role of gut-associated lymphoid tissues in the generation of immunoglobulin-bearing lymphocytes in sheep. Aust J Exp Biol Med Sci.

[B13] Reynaud CA, Mackay CR, Muller RG, Weill JC (1991). Somatic generation of diversity in a mammalian primary lymphoid organ: the sheep ileal Peyer's patches. Cell.

[B14] St Rose SG, Hunter N, Matthews L, Foster JD, Chase-Topping ME, Kruuk LE, Shaw DJ, Rhind SM, Will RG, Woolhouse ME (2006). Comparative evidence for a link between Peyer's patch development and susceptibility to transmissible spongiform encephalopathies. BMC Infect Dis.

[B15] Austbø L, Espenes A, Olsaker I, Press CM, Skretting G (2007). Increased PrP mRNA expression in lymphoid follicles of the ileal Peyer's patch of sheep experimentally exposed to the scrapie agent. J Gen Virol.

[B16] Lechner S, Muller-Ladner U, Renke B, Scholmerich J, Ruschoff J, Kullmann F (2003). Gene expression pattern of laser microdissected colonic crypts of adenomas with low grade dysplasia. Gut.

[B17] Judex M, Neumann E, Lechner S, Dietmaier W, Ballhorn W, Grifka J, Gay S, Scholmerich J, Kullmann F, Muller-Ladner U (2003). Laser-mediated microdissection facilitates analysis of area-specific gene expression in rheumatoid synovium. Arthritis Rheum.

[B18] Judex M, Neumann E, Gay S, Muller-Ladner U (2004). Laser-mediated microdissection as a tool for molecular analysis in arthritis. Methods Mol Med.

[B19] National Center for Biotechnology Information. http://www.ncbi.nlm.nih.gov/blast.

[B20] Moody DE (2001). Genomics techniques: An overview of methods for the study of gene expression. J Anim Sci.

[B21] Booth S, Bowman C, Baumgartner R, Sorensen G, Robertson C, Coulthart M, Phillipson C, Somorjai RL (2004). Identification of central nervous system genes involved in the host response to the scrapie agent during preclinical and clinical infection. J Gen Virol.

[B22] Riemer C, Queck I, Simon D, Kurth R, Baier M (2000). Identification of upregulated genes in scrapie-infected brain tissue. J Virol.

[B23] Baker CA, Lu ZY, Manuelidis L (2004). Early induction of interferon-responsive mRNAs in Creutzfeldt-Jakob disease. J Neurovirol.

[B24] Skretting G, Espenes A, Ulvund MJ, Olsaker I (2004). cDNA representational difference analysis of ileal Peyer's patches in lambs after oral inoculation with scrapie. Biochem Biophys Res Commun.

[B25] Nakagawa H, Koyama K, Murata Y, Morito M, Akiyama T, Nakamura Y (2000). EB3, a novel member of the EB1 family preferentially expressed in the central nervous system, binds to a CNS-specific APC homologue. Oncogene.

[B26] Han J, Zhang J, Yao H, Wang X, Li F, Chen L, Gao C, Gao J, Nie K, Zhou W, Dong X (2006). Study on interaction between microtubule associated protein tau and prion protein. Sci China C Life Sci.

[B27] Brion JP, Fraser H, Flament-Durand J, Dickinson AG (1987). Amyloid scrapie plaques in mice, and Alzheimer senile plaques, share common antigens with tau, a microtubule-associated protein. Neurosci Lett.

[B28] Chernoff YO (2007). Stress and prions: lessons from the yeast model. FEBS Lett.

[B29] Stobart MJ, Parchaliuk D, Simon SL, Lemaistre J, Lazar J, Rubenstein R, Knox JD (2007). Differential expression of interferon responsive genes in rodent models of transmissible spongiform encephalopathy disease. Mol Neurodegener.

